# Murine Monoclonal Antibodies against the Receptor Binding Domain of SARS-CoV-2 Neutralize Authentic Wild-Type SARS-CoV-2 as Well as B.1.1.7 and B.1.351 Viruses and Protect *In Vivo* in a Mouse Model in a Neutralization-Dependent Manner

**DOI:** 10.1128/mBio.01002-21

**Published:** 2021-07-27

**Authors:** Fatima Amanat, Shirin Strohmeier, Wen-Hsin Lee, Sandhya Bangaru, Andrew B. Ward, Lynda Coughlan, Florian Krammer

**Affiliations:** a Graduate School of Biomedical Sciences, Icahn School of Medicine at Mount Sinai, New York, New York, USA; b Department of Microbiology, Icahn School of Medicine at Mount Sinai, New York, New York, USA; c Department of Integrative Structural and Computational Biology, The Scripps Research Institute, La Jolla, California, USA; d University of Maryland School of Medicine, Maryland, USA; St. Jude Children's Research Hospital

**Keywords:** RBD, SARS-CoV-2, monoclonal antibodies

## Abstract

After first emerging in late 2019 in China, severe acute respiratory syndrome coronavirus 2 (SARS-CoV-2) has since caused a pandemic leading to millions of infections and deaths worldwide. Vaccines have been developed and authorized, but the supply of these vaccines is currently limited. With new variants of the virus now emerging and spreading globally, it is essential to develop therapeutics that are broadly protective and bind conserved epitopes in the receptor binding domain (RBD) or the full-length spike protein of SARS-CoV-2. In this study, we generated mouse monoclonal antibodies (MAbs) against different epitopes on the RBD and assessed binding and neutralization of authentic SARS-CoV-2. We demonstrate that antibodies with neutralizing activity, but not nonneutralizing antibodies, lower viral titers in the lungs when administered in a prophylactic setting *in vivo* in a mouse challenge model. In addition, most of the MAbs cross-neutralize the B.1.351 as well as the B.1.1.7 variant *in vitro*.

## INTRODUCTION

Severe acute respiratory syndrome coronavirus 2 (SARS-CoV-2) first emerged in late 2019 in the Hubei province of China, spread rapidly throughout the globe, and has since caused the ongoing coronavirus disease 2019 (COVID-19) pandemic (1, 2). Millions of infections have occurred globally, and over 3.8 million deaths have been caused by this novel coronavirus as of 18 June 2021 according to the World Health Organization. Over a hundred vaccines are currently in clinical development, with three vaccines (BNT162b2 [Pfizer/BioNTech], mRNA-1273 [Moderna], and JNJ-78436735 [Johnson & Johnson]) authorized for use in humans under the emergency use authorization (EUA) mechanism in the United States by the Food and Drug Administration (FDA) and several additional ones approved in Europe, Latin America, and Asia. Furthermore, several monoclonal antibodies (MAbs) and an antiviral, remdesivir, have been authorized for use in humans as therapeutics, and numerous other antivirals are in development (3–6). REGN-COV2, a cocktail of casirivimab and imdevimab, targets the receptor binding domain (RBD) of the spike protein and has been granted EUA (7, 8). Bamlanivimab monotherapy as well as a cocktail of bamlanivimab and etesevimab have also received EUA (9, 10).

SARS-CoV-2, a positive-sense single-stranded RNA virus and member of the *Coronaviridae* family, is closely related to SARS-CoV-1, which caused a major outbreak from 2002 to 2004. Both viruses use the same receptor for entry into host cells, human angiotensin-converting enzyme 2 (hACE2) (11, 12). The RBD, which is part of the spike protein of the virus, can bind to hACE2 and mediate entry, and thus, the spike protein makes for an excellent target for vaccines and therapeutics (13). It has been observed that sera from infected individuals as well as from vaccinated individuals contain a robust level of antibodies against the spike protein and that this serum has neutralizing activity (14–16). Antibodies induced by natural infection with SARS-CoV-2 correlate with protection, and vaccination has been shown to be highly efficacious (15, 17–22). However, it is still crucial to develop therapeutics that can be used to treat individuals who are infected with SARS-CoV-2, particularly those at high risk for severe disease. While MAbs have been developed and approved for use, there remains a significant concern about the virus acquiring mutations that would lead to escape, rendering the MAbs and vaccines inefficient in blocking virus and stopping replication of the virus in the body. Several lineages of SARS-CoV-2 with distinct mutations in the spike protein have emerged over the last year (23). Mutations in the RBD region of the spike protein are a serious concern, as many neutralizing antibodies target the RBD and block entry (24–26). Another region heavily mutated in the new circulating variant viruses is the N-terminal domain (NTD), which is also a target of neutralizing antibodies (27). Hence, the efficacy of vaccines and therapeutics may be compromised as more and more mutations in the NTD and RBD occur and persist in nature (28, 29).

In this study, we isolated and characterized 14 mouse MAbs against the RBD of SARS-CoV-2 and assessed their binding to recombinant RBD and spike protein, as well as tested their ability to neutralize live virus. In addition, we tested if nonneutralizing MAbs can lower viral loads in a mouse challenge model. Due to the new variants of concern which have been detected, we also tested if MAbs can bind mutant RBDs that contain single amino acid changes as well as multiple mutations found in the RBDs of B.1.1.7 (originally detected in the United Kingdom), B.1.351 (originally detected in South Africa), and P.1 (originally detected in Brazil). Lastly, we tested our panel of neutralizing MAbs against a B.1.1.7 virus isolate as well as a B.1.351 virus isolate.

## RESULTS

### Generation of monoclonal antibodies.

After two vaccinations of BALB/c mice with recombinant RBD protein supplemented with poly(I·C) as adjuvant, murine hybridoma technology was used to generate hybridoma cell lines that secreted RBD-specific monoclonal antibodies (30–32). Fourteen hybridoma lines that produced IgGs were isolated and picked (Table 1). Twelve monoclonal antibodies belonged to the IgG1 subclass, while two monoclonal antibodies were from the IgG2a subclass.

**TABLE 1:** Characteristics of MAbs used in this study

MAb	Isotype	RBD ELISA MBC (μg/ml)	Spike ELISA MBC (μg/ml)	IC_50_ (wild-type SARS-CoV-2) (μg/ml)^*a*^	*In vivo* protection
KL-S-1B5	IgG1	0.123457	1.11	ND	No
KL-S-1D2	IgG2a	0.000508	0.00051	0.49	Yes
KL-S-1D11	IgG1	0.000508	2.22	ND	No
KL-S-1E10	IgG1	0.004572	0.0046	ND	No
KL-S-1F7	IgG1	0.000508	0.00051	1.8	Yes
KL-S-1H12	IgG1	0.000508	0.0015	5.1	Yes
KL-S-2A1	IgG1	0.123457	1.11	ND	No
KL-S-2A5	IgG1	0.004572	0.0046	ND	No
KL-S-2C3	IgG2a	0.000508	0.0046	1.1	Yes
KL-S-2F1	IgG1	0.001524	0.0046	ND	No
KL-S-2F9	IgG1	0.000508	0.00051	4.0	Yes
KL-S-2G9	IgG1	0.004572	0.0046	ND	No
KL-S-3A5	IgG1	0.000508	0.00051	ND	No
KL-S-3A7	IgG1	0.000508	0.00051	0.8	Yes

a ND, not detected.

### All antibodies bind the RBD of SARS-CoV-2, and six MAbs neutralize live virus.

**FIG 1:**
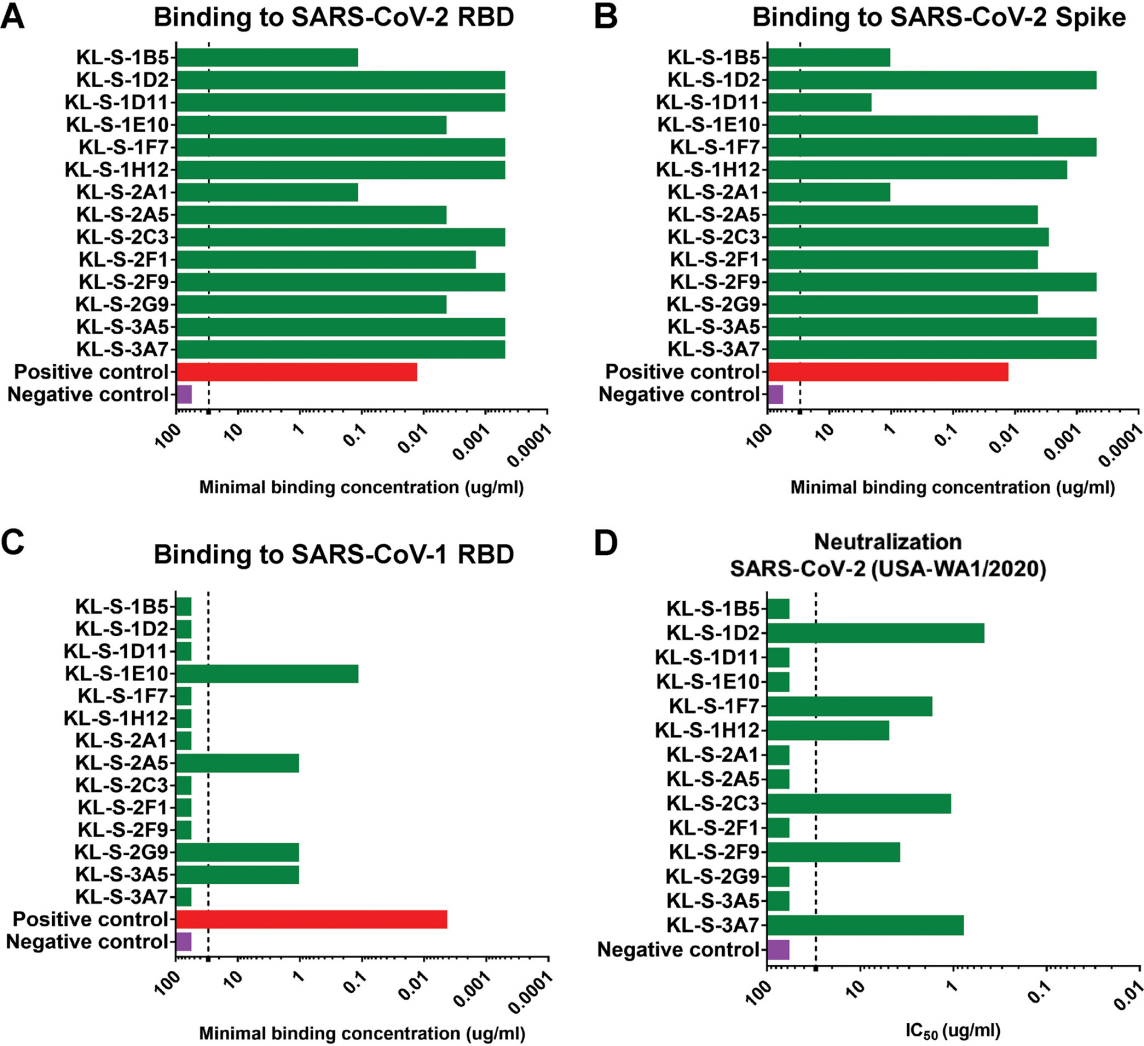
All MAbs bind to the recombinant RBD, and six MAbs neutralize SARS-CoV-2. (A) Binding of all isolated MAbs (*n* = 14) via an ELISA was assessed against recombinant RBD proteins, and the MBC values are shown. The positive control used was a human antibody against the SARS-CoV-1 RBD, CR3022, while the negative control used was a mouse anti-influenza H10 antibody. Binding of all isolated MAbs was also tested by ELISA against the spike protein of SARS-CoV-2 (B) as well as the SARS-CoV-1 RBD (C). (D) The neutralization activities of all MAbs were tested in a microneutralization assay with authentic SARS-CoV-2 (USA-WA1/2020), starting at 30 μg/ml, and subsequent 3-fold dilutions were tested. The cells were stained for the nucleoprotein of SARS-CoV-2, and the IC_50_ values were calculated via a nonlinear regression fit. All experiments were performed in duplicates.

Once all antibodies were purified from the hybridoma supernatant, a standard enzyme-linked immunosorbent assay (ELISA) was performed to assess binding of the monoclonal antibodies to the RBD of SARS-CoV-2 (Fig. 1A), the full spike protein of SARS-CoV-2 (Fig. 1B), and the RBD of SARS-CoV-1 (Fig. 1C). All antibodies bound well to the SARS-CoV-2 RBD, and most had very low minimal binding concentrations (MBCs). Of note, the MBC values for KL-S-1B5 and KL-S-2A1 (0.1 μg/ml) against the SARS-CoV-2 RBD were higher than those for the remaining antibodies, indicating lower affinity. Next, antibodies were tested in an ELISA against the full spike protein of SARS-CoV-2 (Fig. 1B). It is interesting to note that while most MAbs bound well, with low MBC values, KL-S-1B5, KL-S-1D11, and KL-S-2A1 had higher MBC values against spike than against the RBD of SARS-CoV-2. It is possible that the epitope of these antibodies is partially occluded on the full spike protein compared to the RBD protein when expressed alone. To determine if antibodies were cross-reactive to the RBD of SARS-CoV-1, an ELISA was performed (Fig. 1C). Most MAbs did not bind the RBD of SARS-CoV-1; exceptions were KL-S-1E10, KL-S-2A5, KL-S-3G9, and KL-S-3A5. To assess the functionality of the MAbs, all 14 MAbs were tested in a microneutralization assay with authentic SARS-CoV-2 for their ability to neutralize live virus (Fig. 1D). Six MAbs (43%) neutralized live virus well, with low 50% inhibitory concentration (IC_50_) values (0.1 to 1 μg/ml), indicating that low concentrations are capable of blocking virus entry and/or replication. Notably, KL-S-1D2 and KL-S-3A7 have extraordinarily low IC_50_ values, at below 1 μg/ml.

### Antibodies can reduce viral titers *in vivo* in a mouse challenge model.

**FIG 2:**
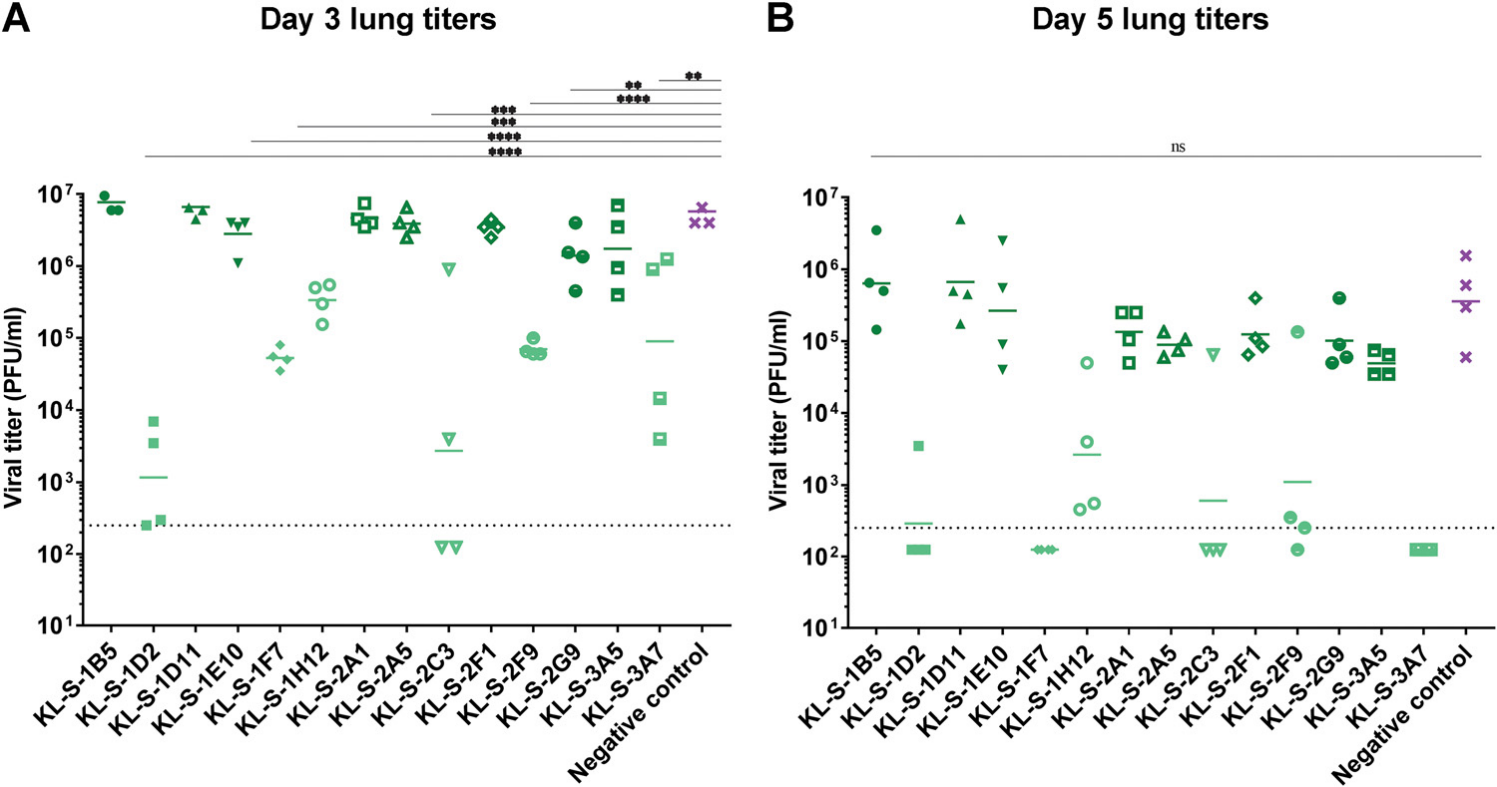
Only neutralizing MAbs lower viral loads *in vivo* in an AdV-hACE2 mouse challenge model. The protective efficacies of the MAbs were assessed *in vivo* in a prophylactic setting. Mice were administered 2.5 × 10^8^ PFU per mouse of AdV-hACE2, and 5 days later, mice were administered each MAb (*n* = 4) at 10 mg/kg and challenged 2 hours later with 10^5^ PFU of SARS-CoV-2. Viral titers in the lungs were assessed at day 3 (A) and day 5 (B) post infection via a plaque assay. Mice in the negative-control group received a mouse anti-influenza H10 antibody. Statistical significance was determined via a one-way ANOVA test with multiple comparisons (****, *P* ≤ 0.0005; ***, *P* ≤ 0.005; **, *P* ≤ 0.05; ns, not significant).

To further study the biological functionality of these MAbs, all MAbs were tested *in vivo*. Hence, an animal model was utilized to test if antibodies are able to inhibit viral replication and thus reduce virus titers in the lung. Since mouse ACE2 does not facilitate the entry of SARS-CoV-2, an adenovirus expressing the human ACE2 gene (AdV-hACE2) was used to transduce mice (33, 34). Five days after an adenovirus treatment, monoclonal antibodies were administered at 10 mg/kg of body weight 2 h prior to infection with SARS-CoV-2 and lungs were collected on day 3 and day 5 postinfection to assess viral titers via a plaque assay. Only neutralizing MAbs were able to confer a protective benefit and lowered viral titers in the lungs (Fig. 2A and B). On day 3, several groups had significantly lower virus titers in their lungs than the control group. The effect was most pronounced for KL-S-1D2 and KL-S-2C3. KL-S-1F7-, KL-S-1H12-, and KL-S-2F9-treated animals which had approximately 2 logs less virus in their lungs than animals in the negative-control group on day 3 (Fig. 2A). The negative control used here was an irrelevant purified antibody that binds to influenza virus H10 hemagglutinin (31). On day 5, groups that received the six neutralizing MAbs had little-to-no virus in their lungs (Fig. 2B). Only one mouse in the KL-S-2C3 group and one mouse in the KL-S-2F9 group showed residual virus in their lungs. None of the nonneutralizing antibodies conferred any protective benefit. Of note, all of the non-neutralizing MAbs belonged to the IgG1 isotype.

### Neutralizing antibodies eliminate the viral presence in the lungs, and few differences were found between the groups in terms of lung pathology.

**FIG 3:**
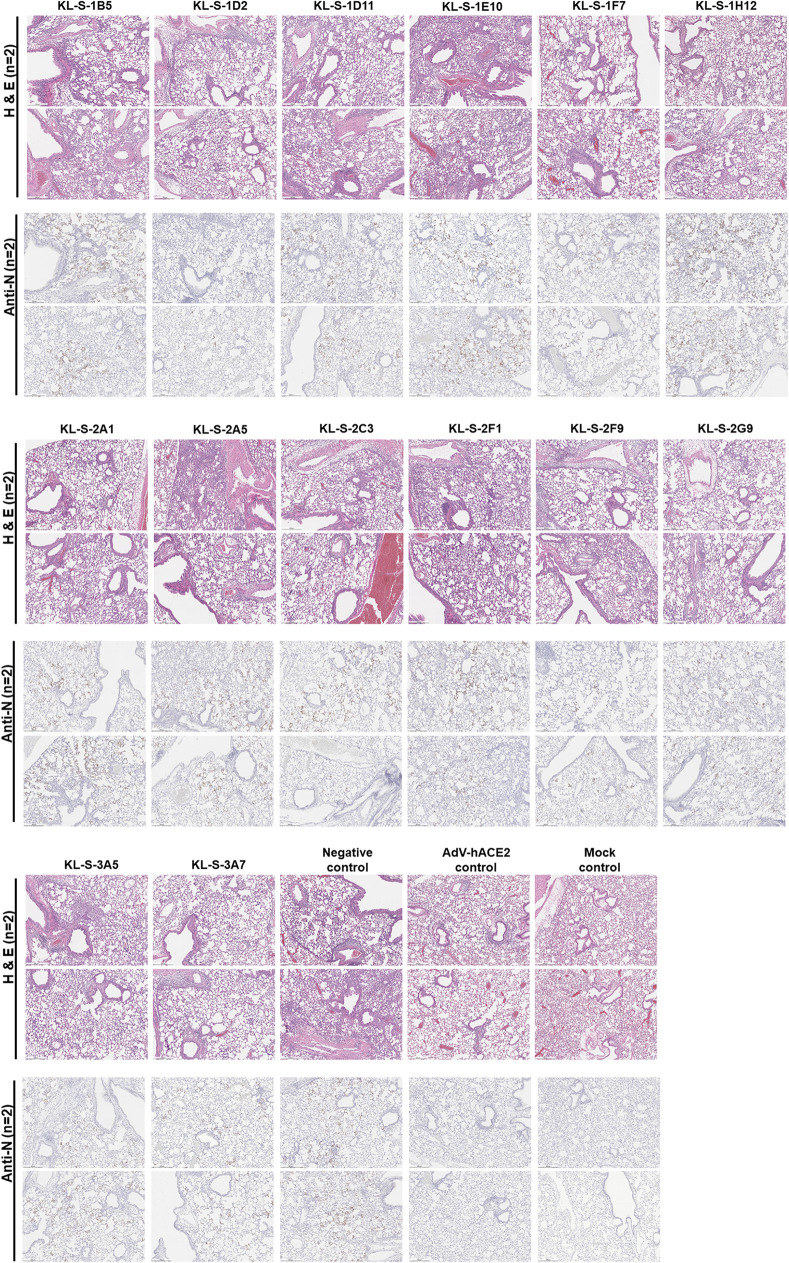
Histopathological effects after MAb administration and challenge in the lungs. (A) In order to assess if antibodies can have any negative immunopathological effects, lungs were harvested from each antibody group (*n* = 2), as shown. Two mice received only the AdV-hACE2, while two mice were naive. (B) An antinucleoprotein antibody (Anti-N) was used to check for the presence of virus in the lungs. Scale bar = 500 μm.

In addition to assessing viral titers in the lungs in a prophylactic setting, we also wanted to test if MAbs can protect from inflammation and/or tissue damage in the lungs or lead to enhanced disease, which has been noted for SARS-CoV-1 (35). Lungs were harvested on day 4 postinfection from all the antibody groups (*n* = 2) and subjected to histopathological analysis (HistoWiz), such as hematoxylin and eosin (H&E) staining, as well as immunohistochemistry (IHC) using an antibody specific for the nucleoprotein of SARS-CoV-2. A 5-point grading scheme that took into account six different parameters (perivascular inflammation, bronchial/bronchiolar epithelial degeneration/necrosis, bronchial/bronchiolar inflammation, intraluminal debris, alveolar inflammation, and congestion/edema) was utilized to score lung sections. Interestingly, mice from all groups treated with antibodies displayed some pulmonary histopathological lesions of interstitial pneumonia (Fig. 3). This may be a result of the high dose (10^5^ PFU per mouse) of SARS-CoV-2 used. The group that received only the AdV-hACE2 exhibited lesions of perivascular, peribronchiolar, and alveolar inflammation to some degree and had much lower scores than the antibody groups that received AdV-hACE2 plus SARS-CoV-2 (see Fig. S1 in the supplemental material). This demonstrates that there is some mild inflammation associated with the intranasal administration of AdV-hACE2 alone, and this has been observed in earlier studies (33). Histopathological lesions were uniformly absent in the naive mock group that received no treatment. Clinical scores were slightly higher for groups receiving KL-S-1E10, KL-S-2A5, and KL-S-3A5 than for the negative controls. Both of these antibodies are nonneutralizing, but this may be a result of external variables (e.g., cage-to-cage variability, slight differences in mouse age [6- to 8-week-old mice were used], etc.) in the experimental setup.

10.1128/mBio.01002-21.1 FIG S1 Clinical scores from the lung sections from each antibody group (*n* = 2) are shown. All sections were scored by an independent veterinary pathologist. We used a negative-control group and one control group, which received only AdV-hACE2, while naive mice were also included as a mock control. Download FIG S1, PDF file, 0.3 MB. Copyright © 2021 Amanat et al. 2021 Amanat et al. https://creativecommons.org/licenses/by/4.0/ This content is distributed under the terms of the Creative Commons Attribution 4.0 International license.

In terms of the nucleoprotein staining via IHC, it became clear that all neutralizing MAbs except KL-S-1H12 blocked viral replication, and thus, very little staining for nucleoprotein was observed on day 4. This correlates well with the lung titers found in Fig. 2, as antibodies blocked viral replication and lowered viral load in the lungs.

### Antibodies maintain binding to most variant RBDs.

**FIG 4:**
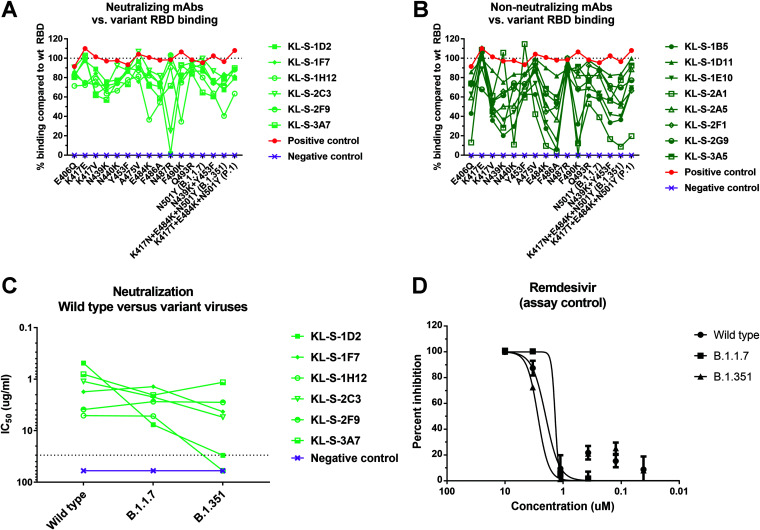
Binding of MAbs to variant RBDs as well cross-neutralization of B.1.1.7 and B.1.351 variant viruses. (A, B) All 14 antibodies were tested in ELISAs for binding to RBDs that contain single or multiple mutations found in new variants. The line at 100% indicates binding to the wild-type (wt) RBD, and binding to each mutant RBD is graphed as a percentage of the binding to the wild-type RBD. A negative-control MAb, anti-influenza H10, was run against all the RBDs to ensure that there is no unspecific binding. A positive control, antihistidine antibody, was used to ensure that the RBD proteins, which have a hexa-histidine tag, are coated properly. (A and B) Neutralizing MAbs (A) and nonneutralizing MAbs (B) are shown separately. (C) A microneutralization assay was performed to test whether the neutralizing MAbs can also neutralize new variant viruses, B.1.1.7 and B.1.351. IC_50_ values of the six neutralizing MAbs for each virus are shown. (D) Remdesivir was also run on a neutralization assay against the wild-type virus, the B.1.1.7 isolate, and the B.1.351 isolate.

Several variants of concern (VOC) with mutations in the RBD are circulating. In addition, studies on MAb escape, *in vitro* evolution of the spike protein, and clinical isolates from immunosuppressed patients have reported a variety of single mutations that may influence antibody binding to the RBD (28, 36–39). We expressed a number of these RBD variants, including those with N439K, Y453F, E484K, and N501Y (B.1.1.7), and the RBDs of B.1.351 and P.1 and performed ELISAs on them using our MAbs. Such an analysis can point towards the epitope of the antibody or a single amino acid that is crucial for binding. The neutralizing MAbs and nonneutralizing MAbs are shown separately (Fig. 4A and B). KL-S-1D2 maintained binding to all RBDs but lost complete binding to the N487R RBD (Fig. 4A). This may be a crucial amino acid for the antibody to maintain its footprint on the RBD. KL-S-2C3 bound to the N487R RBD at only 30% of the level it bound to the wild-type RBD. KL-S-1H12 bound to the E484K, F486A, and F490K RBDs and the B.1.351 RBD at levels approximately ≤50% of the level it bound to the wild-type RBD (Fig. 4A). KL-S-1D11, KL-S-1F7, KL-S-2F9, KL-S-2G9, and KL-S-3A7 were able to bind all mutant RBDs at levels that were ≥50% of their levels of binding to the wild-type RBD (Fig. 4A). KL-S-KL-S-2A1’s levels of binding to the E406Q, N440K, E484K, F490K, B.1.1.7, P.1, and B.1.351 RBDs were <50% of its level of binding to the wild-type RBD (Fig. 4B). The ability to bind all RBDs may be a function of antibody affinity, which, when high, can allow the antibody to maintain its footprint. In general, neutralizing MAbs had comparable levels of binding to both the wild-type RBD and most mutant RBDs. To ensure that the ELISA setups were comparable across mutants, an antihistidine antibody was used as a positive control.

### Four MAbs maintain neutralizing activity to the B.1.351 virus, while all six MAbs neutralize the B.1.1.7 virus.

Since binding may not be directly related to neutralization, we wanted to assess if antibodies that were generated by vaccination of mice with the wild-type RBD can neutralize new variant viruses. These variant viruses carry mutations in the RBD and can escape neutralization by monoclonal antibodies easily if their native epitope has been disrupted (6, 29). Notably, all antibodies that neutralized wild-type SARS-CoV-2 were also able to neutralize the B.1.1.7 virus (although with KL-S-1D2 strongly losing potency), and this is not surprising, as the only mutation present in the RBD of this virus is N501Y (Fig. 4C). However, KL-S-1D2 and KL-S-1H12 completely lost neutralizing activity toward the B.1.351 virus (Fig. 4C). KL-S-1D2 bound the RBD of B.1.351 at around 70% of the level that it bound to the wild-type RBD, but the loss of neutralization may be due to the epitope being presented on the full spike differently from on the RBD alone, leading to a loss of affinity. KL-S-1H12 showed much lower binding to the E484K RBD (60%) as well as the B.1.351 RBD (50%), and this lower binding capability might be the reason for the loss of neutralization. The remaining four MAbs, KL-S-1F7, KL-S-2C3, KL-S-2F9, and KL-S-3A7, maintained strong neutralizing activity against B.1.351 (Fig. 4C). Remdesivir was used as a positive control for the neutralization assay against the three viruses (Fig. 4D).

### Three antibodies block ACE2 from binding the RBD.

**FIG 5:**
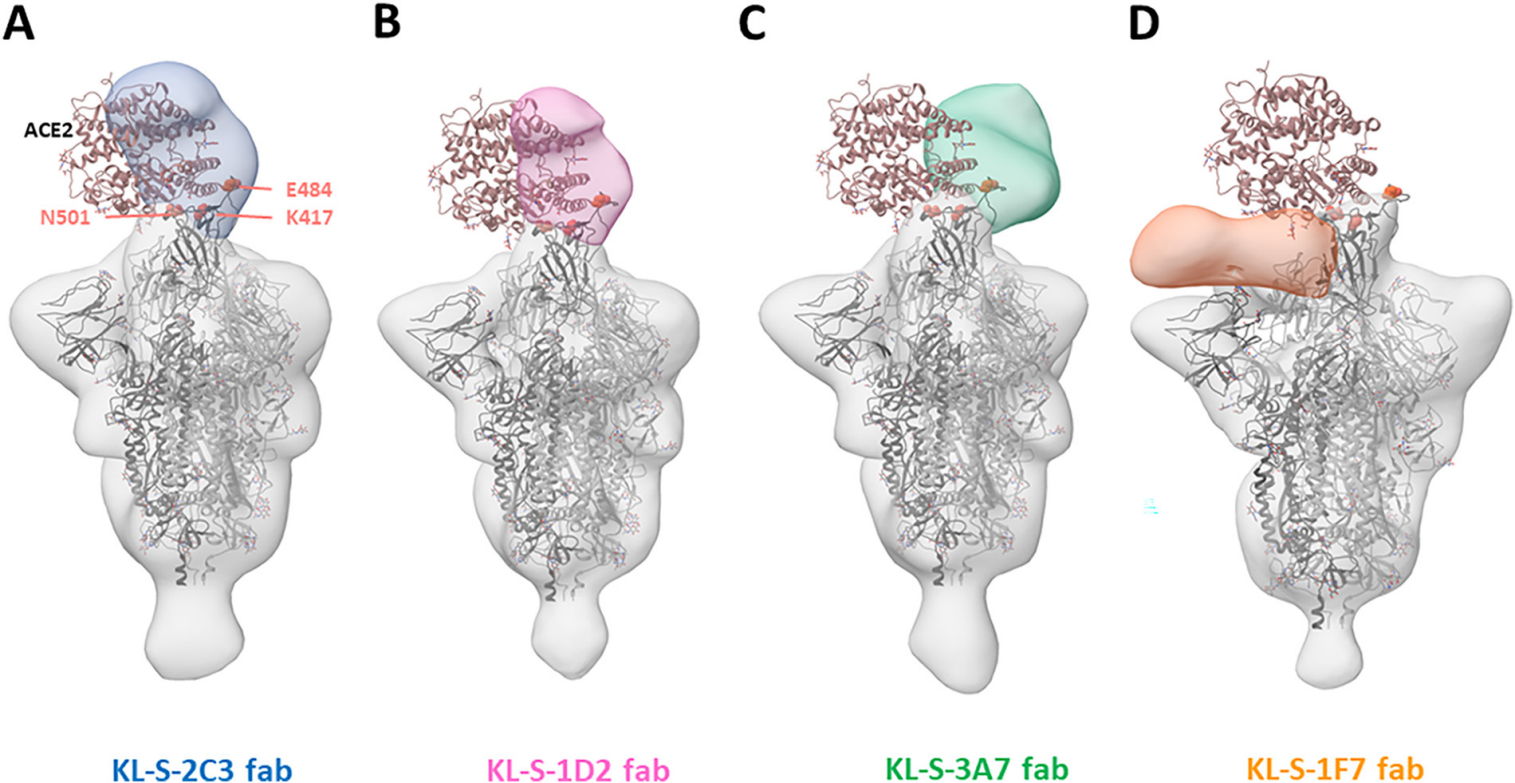
Negative-stain EM analysis of Fabs bound to the SARS-CoV-2 spike trimer. Three-dimensional reconstructions of the Fabs KL-S-2C3 (blue) (A), KL-S-1D2 (pink) (B), KL-S-3A7 (green) (C), and KL-S-1F7 (orange) (D) bound to the stabilized SARS-CoV-2 spike trimer (gray). A model of the spike trimer bound to the ACE2 receptor (PDB accession no. 7KNB) is fit into each density to illustrate their potential for blocking receptor binding.

To study where the neutralizing antibodies bind on the RBD, structural analysis was performed, and negative-stain three-dimensional reconstructions were obtained for four of the neutralizing antibodies (Fig. 5). KL-S-1H12 and KL-S-2F9 did not form stable complexes and were therefore not amenable to image analysis, which may be a result of pH changes or the low affinity/avidity of Fab. KL-S-2C3 (Fig. 5A), KL-S-1D2 (Fig. 5B), and KL-S-3A7 (Fig. 5C) overlap the ACE2 binding site, consistent with blockade of ACE2 binding to the RBD. The antibodies approach at different angles and appear to belong to class 1 (KL-S-3A7 and KL-S-1D2) and class 2 (KL-S-2C3) RBD epitopes (40). KLS-1F7 binds low on the RBD to an epitope similar to the S309 epitope (Fig. 5D) (41).

## DISCUSSION

The RBD of the spike protein of SARS-CoV-2 is relatively plastic and can tolerate extensive mutations, at least *in vitro* (7, 42). The plasticity of the RBD is alarming because extensive changes in the RBD may reduce the efficacies of current vaccines, and additional booster vaccinations with updated vaccines may be needed for protection in the future (28, 29). We tested all 14 isolated MAbs for binding to a whole panel of mutant RBDs. While some MAbs lost binding for many mutant RBDs, other MAbs maintained binding against the majority of mutant RBDs that we used, which are also found in nature. However, binding was not in all cases directly linked to neutralization. All of the neutralizing MAbs maintained binding and neutralizing activity to B.1.1.7 (N501Y) relatively well. However, two MAbs, KL-S-1D2 and KL-S-1H12, lost neutralizing activity against B.1.351, and KL-S-1D2 showed only a relatively small reduction in binding to E484K and B.1.351 RBDs. KL-S-1H12 showed a stronger reduction in binding, which agrees better with the loss of neutralizing activity. Other hot spots for a loss of binding for neutralizing antibodies included amino acid positions 487 and 490.

For four of the neutralizing MAbs, low-resolution structures were solved using single-particle electron microscopy (EM). They included KL-S-1D2, which lost neutralizing activity to B.1.351. Our low-resolution structural analysis precludes interpretation of molecular interactions, but the reduction or loss of neutralization of B1.1.7 and B.1.351 by KL-S-1D2 suggests that N501 and E484 form critical interactions.

Fc-Fc receptor (FcR) interactions may be contributing to the protection that neutralizing antibodies confer *in vivo*. This has been shown for other MAbs developed against SARS-CoV-2, which showed less protection *in vivo* when the Fc was mutated (19). While the role of Fc-Fc receptor interaction-based effector functions for SARS-CoV-2-targeting antibodies is not fully understood yet, it is likely that these effector functions contribute to protection (43). This has also been demonstrated for influenza viruses as well as ebolaviruses (44, 45). We tested all isolated MAbs for their protective effect in a mouse model and found that the only correlation with protection was neutralizing activity, while nonneutralizing antibodies had no effect. However, there is an important caveat that needs to be discussed for this experiment. All nonneutralizing antibodies that we isolated were of the IgG1 subtype, which in mice is known to have low affinity for activating FcRs. This is in contrast to murine IgG2a and IgG2b, which have high affinity for these FcRs. Therefore, we can only conclude that non-Fc-FcR-based interactions do not contribute to protection by nonneutralizing antibodies. In fact, the two antibodies that provided the best protection, especially on day 3, KL-S-1D2 and KL-S-2C3, are both of the IgG2a subtype. While KL-S-1D2 showed the best *in vitro* neutralization of all isolated MAbs, which may cause this phenotype, KL-S-2C3’s *in vitro* activity was lower but still showed stronger activity *in vivo* than those of other MAbs. This might be seen as evidence that Fc-FcR interactions, especially engagement with activating FcRs, are an important component of protection. Of note, the vast majority of antibodies induced in humans to SARS-CoV-2 spike by natural infection or vaccination are IgG1, and in humans, unlike in mice, IgG1 has strong affinity for activating FcRs (46).

One limitation of our study was the assessment of the *in vivo* efficacy of the MAbs only in a prophylactic setting. Due to the limitations of the mouse model used, we were unable to test the MAbs *in vivo* in a therapeutic setting where each MAb would be administered postinfection. It will be important to assess if these antibodies can lower viral loads *in vivo* after infection has occurred, and we hope to further test this in future studies in different animal models.

In summary, we describe several antibodies to the SARS-CoV-2 RBD that maintain strong neutralizing activity against the B.1.1.7 as well as the B.1.351 variant. These MAbs, if humanized, may be further developed into “variant-resistant” therapeutics.

## MATERIALS AND METHODS

### Cells and viruses.

Vero.E6 cells (ATCC CRL-1586) were maintained in Dulbecco’s modified Eagle’s medium (DMEM; Life Technologies), which was supplemented with 10% fetal bovine serum (FBS; Corning) as well as antibiotics (100 units/ml penicillin–100 μg/ml streptomycin [Pen-Strep; Gibco]), and buffer solution (1 M 4-(2-hydroxyethyl)-1-piperazineethanesulfonic acid [HEPES]; Gibco). SARS-CoV-2 was exclusively handled in a biosafety level 3 (BSL3) facility and passaged in Vero.E6 for 3 days, and the supernatant from infected cells was clarified via centrifugation at 1,000 × *g* for 5 min. The titers of virus stocks were determined in Vero.E6 cells as well.

### Generation of monoclonal antibodies.

All animal work was performed by adhering to institutional regulations as well as Institutional Animal Care and Use Committee (IACUC) guidelines. Six- to eight-week-old female mice (Jackson Laboratory) were immunized with 3 μg of the purified RBD of SARS-CoV-2 mixed with 10 μg of poly(I·C) (InvivoGen) twice at 3-week intervals (30, 46). All immunizations were administered via the intramuscular route. Finally, mice were immunized again with 100 μg of the RBD along with 10 μg of poly(I·C). Three days later, the mouse was sacrificed and the spleen was extracted in a sterile manner. The splenocytes were washed with phosphate-buffered saline (PBS; Life Technologies) and then fused with SP2/o myeloma cells (ATCC) using polyethylene glycol (Sigma-Aldrich catalog no. P7181). Hybridoma supernatants were screened in an enzyme-linked immunosorbent assay (ELISA) as described in the section below. Desirable hybridomas that secreted IgG were selected and expanded. Supernatants was collected at the end, filtered using a 0.22-μm filter, and then purified via protein G-Sepharose (GE Health) using gravity flow (31, 44, 45, 47–51).

### ELISA.

Ninety-six-well, flat-bottom, Immulon 4 HBX plates (Thermo Scientific) were coated overnight at 4°C with 50 μl/well of a 2-μg/ml solution of each respective protein in phosphate-buffered saline (PBS). The next day, the coating solution was discarded. One hundred microliters per well of 3% nonfat milk prepared in PBS containing 0.1% Tween 20 (T-PBS; Fisher Bioreagents) was added to the plates for 1 h at room temperature (RT) to block the plates. Antibody dilutions were prepared in 1% nonfat milk in T-PBS. The starting concentration used for each antibody was 30 μg/ml, and 3-fold dilutions were subsequently prepared. After the blocking solution had been on the plates for 1 h, the antibody dilutions were added for 1 h at RT. Next, the plates were washed thrice with 250 μl per well of T-PBS. The secondary solution was also prepared in 1% nonfat milk in T-PBS. For mouse antibodies, anti-mouse IgG conjugated to horseradish peroxidase (Rockland) was used at a dilution of 1:3,000. For human antibodies, anti-human IgG Fab was used at the same dilution. After 1 h, plates were washed thrice with 250 μl per well of T-PBS and developing solution was prepared using SigmaFast *o*-phenylenediamine dihydrochloride (OPD). One hundred microliters per well of developing solution was added for exactly 10 min, after which the reaction was stopped by addition of 50 μl per well of 3 M HCl (Fisher Bioreagents). Optical density at 490 nm was measured using a plate reader, BioTek Synergy H1. All data were analyzed using GraphPad Prism 7. An antihistidine antibody was used for ELISAs as a positive control (TaKaRa; catalog no. 631212).

### Microneutralization assay.

All antibodies were tested for neutralization capability in a neutralization assay with authentic SARS-CoV-2 isolate USA-WA1/2020 (BEI Resources NR-52281), isolate hCoV-19/South Africa/KRISP-K005325/2020 (BEI Resources NR-54009), and hCoV-19/England/204820464/2020 (BEI Resources NR-54000) in the BSL3 facility. All viruses were obtained from BEI Resources and propagated in Vero.E6 cells. Twenty thousand Vero.E6 cells were seeded in a 96-well cell culture plate and used the next day. Antibody dilutions were prepared starting at 30 μg/ml, and subsequent 3-fold dilutions were prepared. The protocol has been described previously (34, 46, 52, 53). Cells were stained for the nucleoprotein and quantified. Percent inhibition was calculated, and IC_50_s were obtained (52). All viruses were subjected to deep sequencing to ensure that no mutations had taken place in culture. The polybasic cleavage site changed to WRAR in the B.1.351 strain during passage in cell culture (which is known for this virus from BEI Resources), and no other unexpected mutations occurred.

### *In vivo* mouse challenge studies.

All work with SARS-CoV-2 was performed in a BSL3 facility. Six- to eight-week-old female BALB/c mice (Jackson Laboratory) were administered an adenovirus expressing human ACE2 (AdV-hACE2) via the intranasal route at 2.5 × 10^8^ PFU per mouse in a final volume of 50 μl. Five days later, each respective antibody was administered via the intraperitoneal route at 10 mg/kg in a 100-μl volume. Two hours later, mice were infected with 10^5^ PFU of SARS-CoV-2 intranasally. Mice were humanely sacrificed on day 3 and day 5 to assess the viral titers in the lungs. Lungs were homogenized using a BeadBlaster 24 (Benchmark) homogenizer. Each lung homogenate was tested in a classical plaque assay as described previously (44, 47).

### Plaque assay.

To assess viral titer in the lungs, each homogenate was diluted in 1× minimal essential medium (10× MEM; Life Technologies) supplemented with glutamine, 35% bovine serum albumin (BSA; MP Biomedicals), antibiotics, and HEPES as described previously (46, 54). Three hundred thousand Vero.E6 cells were seeded per well in a 12-well cell culture plate and used the next day, when the cells were approximately 90% confluent. Medium was removed from cells, and dilutions were added to the plates and incubated in a 37°C incubator for 1 h. Next, the virus dilutions were removed, and cells were overlaid with 2% Oxoid agar mixed with 2× MEM. After 3 days, cells were fixed with 10% paraformaldehyde (Polysciences) for 24 h and stained with antispike antibodies, and plaques were counted.

### Histology and IHC.

Mice were administered anesthesia and euthanized by exsanguination of the femoral artery. Lungs were inflated and flushed with 10% formaldehyde by injecting a needle through the trachea on day 4 postinfection with SARS-CoV-2. Fixed lung samples were sent for processing to a commercial company, HistoWiz. Sections were analyzed, images were taken, and sections were also scored by a pathologist. Scores were assigned by the pathologist based on six parameters, as mentioned in Results. Both H&E staining and immunohistochemistry (IHC) were performed. An anti-SARS-CoV nucleoprotein antibody (Novus Biologicals; catalog no. NB100–56576) was used for IHC.

### Expression and purification of recombinant spike proteins for electron microscopy.

The SARS-CoV-2 spike construct used for EM studies contains the mammalian-codon-optimized gene encoding residues 1 to 1208 of the spike protein, followed by a C-terminal T4 fibritin trimerization domain, an HRV3C cleavage site, and an 8×His tag subcloned into the eukaryotic expression vector pcDNA3.4. Amino acid mutations were introduced in the S1/S2 cleavage site (RRAR to GSAS), along with other stabilizing mutations, including the HexaPro mutations (55). The spike trimers were expressed and purified as described previously (18).

### Negative-stain EM sample preparation and data collection.

Spike protein was complexed with purified Fab at a 3× molar excess per trimer and incubated for 30 min at room temperature. Complexes were diluted to 0.02 mg/ml in Tris-buffered saline (TBS), and 3 μl was applied to a 400 mesh Cu grid, blotted with filter paper, and stained with 2% uranyl formate for 30 s. Images were collected on a Tecnai Spirit microscope operating at 120 kV with an FEI Eagle charge-coupled device (CCD; 4k) camera. Particles were picked using DoGpicker, and three-dimensional classification was done using RELION 3.0 (56, 57).

### Statistical analyses.

All data were analyzed using GraphPad Prism 7.0. MBC values were calculated as the last dilution of antibody that gave a signal above average blank values plus 3 times the standard deviation. One-way analysis of variance (ANOVA) with multiple comparisons was used to calculate statistical significance, also in Prism 7.0.
