# Supplementary figures and images for: Murine Monoclonal Antibodies against the Receptor Binding Domain of SARS-CoV-2 Neutralize Authentic Wild-Type SARS-CoV-2 as Well as B.1.1.7 and B.1.351 Viruses and Protect In Vivo in a Mouse Model in a Neutralization-Dependent Manner
Source: mBio. 2021 Jul 27;12(4):e01002-21. doi: 10.1128/mBio.01002-21 (PMC8406178; doi:10.1128/mBio.01002-21)

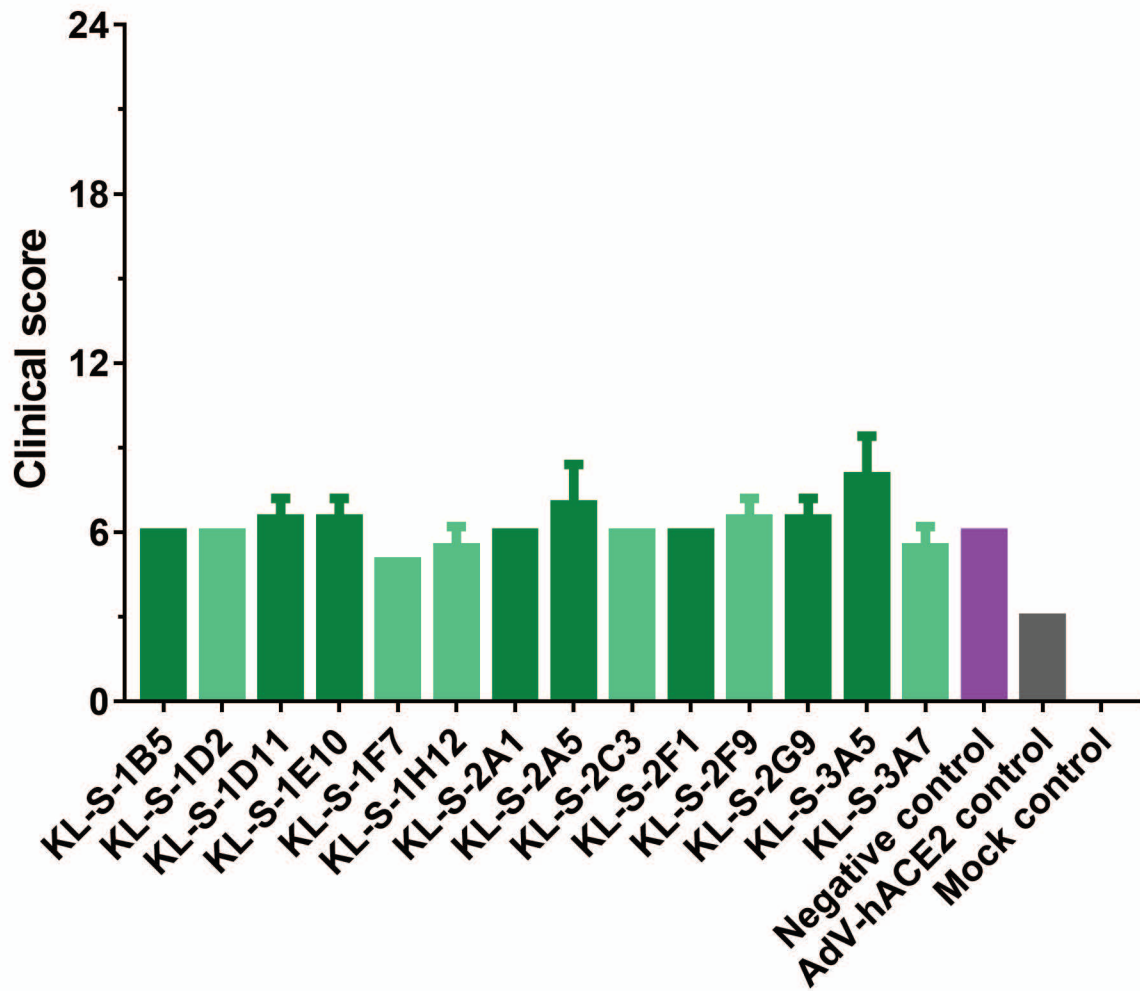

Supplement: FIG S1 [file mbio.01002-21-sf001.pdf]
